# Supplementary material for: Infection Risk in Patients with Dermatomyositis Associated with Anti-MDA5 Antibodies: A Historical Cohort Study
Source: Biomedicines. 2022 Dec 8;10(12):3176. doi: 10.3390/biomedicines10123176 (PMC9776099; doi:10.3390/biomedicines10123176)
Supplement: Supplementary file 1 [file biomedicines-10-03176-s001.zip › biomedicines-2050907-supplementary.pdf]

*Supplementary Materials*

# Infection Risk in Patients with Dermatomyositis Associated with Anti-MDA5 Antibodies: A Historical Cohort Study

**Anne-Claire Billet, Thomas Barba, Frédéric Coutant, Nicole Fabien, Laurent Perard, Pascal Sève, Jean C. Lega, Cécile-Audrey Durel, Laure Gallay and Arnaud Hot**

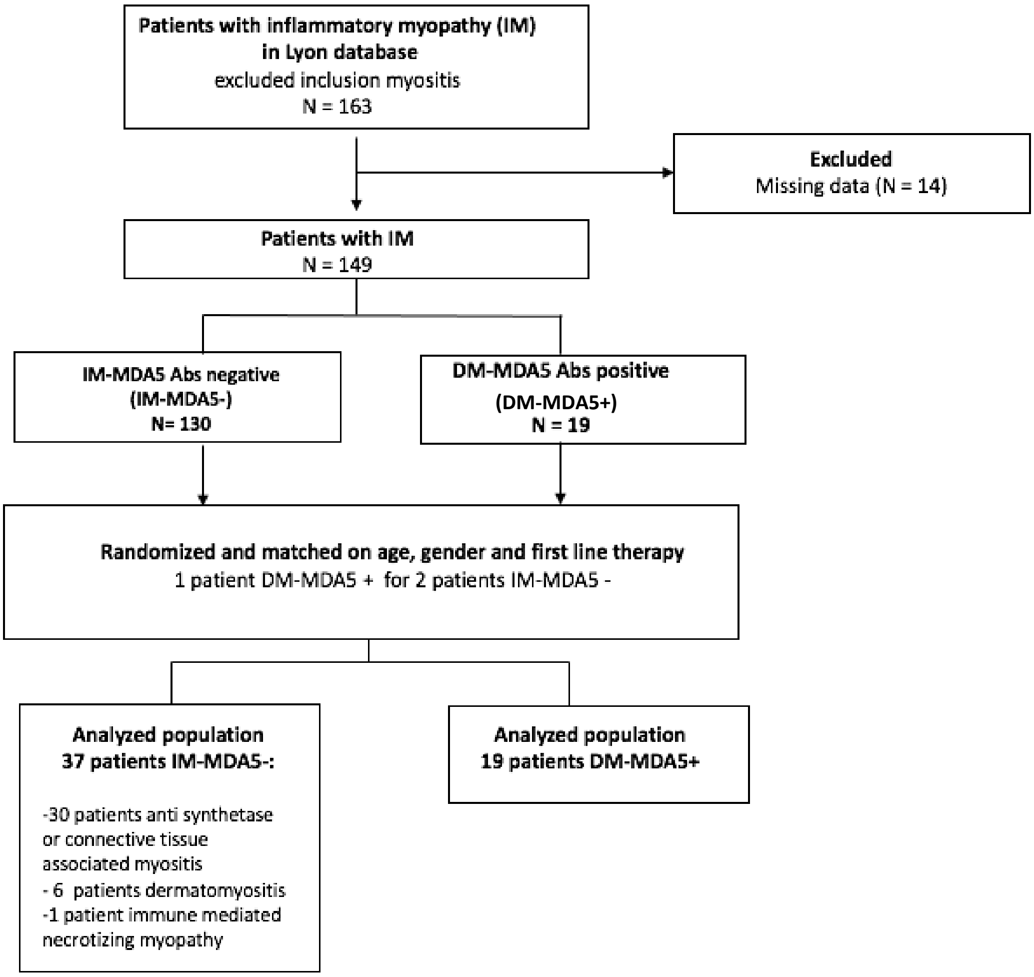


**Figure S1.** Flow chart.

**Table S1.** Immunosuppressive regimens.

| | Patients MDA 5 positive (n = 19) | Patients MDA 5 negative (n = 37) | *p*-value |
|---|---|---|---|
| **Corticosteroids (CS)** | | | |
| Bolus, n (%) | 6 (32) | 2 (5) | 0.02 |
| Initial dose, mg/kg | 0.95 ± 0.12 | 0.95 ± 0.25 | 0.94 |
| Maintenance dose, mg/day | 9.97 ± 15.9 | 5.85 ± 7.7 | 0.33 |
| **First line therapy** | | | |
| **CS alone** | 6 (32) | 12 (33) | - |
| **DMARDs** | 5 (26) | 10 (27) | - |
| Methotrexate | 4/5 | 3/10 | - |
| Mycophenolate mofetil | 1/5 | 5/10 | - |
| Azathioprine | 0/5 | 2/10 | - |
| **IVIG** | 3 (16) | 6 (16) | - |
| **DMARDs and IVIG** | 2 (11) | 4 (11) | - |
| Methotrexate | 1/2 | 0/4 | - |
| Mycophenolate mofetil | 1/2 | 2/4 | - |
| Azathioprine | 0/2 | 2/4 | - |
| **Others** | | | |
| Cyclophosphamide | 2 (11) | 4 (11) | - |
| Rituximab | 1 (5) | 1 (3) | - |

DMARDS: conventional synthetic disease modifying anti-rheumatic drug; CST: corticosteroids; IT: immunosuppressive therapy.

**Table S2.** Univariate analyses of risk factors of SIC.

| Variable | No. of Patients | Univariate | | |
|---|---|---|---|---|
| | | HR | 95% CI | p-value |
| **Demography** | | | | |
| **Gender** | | | | |
| Female | 27 | 1.00 | Reference | |
| Male | 29 | 2.8 | (1.03–7.57) | 0.04 |
| **Age at diagnosis** | 56 | 1.03 | (0.99–1.06) | 0.13 |
| **Comorbidities** | | | | |
| **Diabetes** | | | | |
| No | 51 | 1.00 | Reference | |
| Yes | 5 | 2.99 | (0.86–10.44) | 0.13 |
| **Active smoking/stopped <3 years** | | | | |
| No | 44 | 1.00 | Reference | |
| Yes | 12 | 1.59 | (0.56–4.5) | |
| **Cancer** | | | | |
| No | 47 | 1.00 | Reference | |
| Yes | 9 | 3.97 | (1.46–10.82) | 0.01 |
| **Clinical – Biological Factors** | | | | |
| No | 46 | 1.00 | Reference | |
| Yes | 8 | 3.02 | (1.30–8.08) | 0.04 |
| **Ground glass opacities** | | | | |
| No | 37 | 1.00 | Reference | |
| Yes | 18 | 0.24 | (0.05–1.03) | 0.02 |
| **Pulmonary fibrosis** | | | | |
| No | 38 | 1.00 | Reference | |
| Yes | 17 | 1.41 | (0.55–3.65) | 0.48 |
| **Restrictive disorder** | | | | |
| No | 22 | 1.00 | Reference | |
| Yes | 13 | 0.79 | (0.25–2.46) | 0.68 |
| **Deglutition disorders** | | | | |
| No | 48 | 1.00 | Reference | |
| Yes | 8 | 1.26 | (0.36–4.35) | 0.72 |
| **Ig G (g/L)** | 34 | 1.12 | (0.94–1.33) | 0.22 |
| **Autoimmune Status** | | | | |
| **MDA5 antibodies** | | | | |
| No | 37 | 1.00 | | |
| Yes | 19 | 7.08 | (2.50–20.04) | <0.001 |
| **Prophylaxis** | | | | |
| **Pneumocystis prophylaxis** | | | | |
| No | 34 | 1.00 | Reference | |
| Yes | 20 | 1.7 | (0.67–4.34) | 0.27 |
| **Pneumococcal vaccination** | | | | |
| No | 30 | 1.00 | Reference | |
| Yes | 21 | 0.74 | (0.28–1.98) | 0.54 |
| **Immunosuppressive Regimens** | | | | |
| **Corticosteroids (CS)** | | | | |
| Initial dose (mg/kg) | 49 | 0.98 | (0.11–8.69) | 0.99 |
| Maintenance dose (mg/day) | 47 | 1.06 | (1.02–1.10) | 0.01 |
| **DMARDs** | | | | |
| **Methotrexate** | | | | |
| No | 49 | 1.00 | Reference | |

| | | | | |
|---|---|---|---|---|
| Yes | 7 | 1.77 | (0.70–4.48) | 0.23 |
| **Mycophenolate mofetil** | | | | |
| No | 50 | 1.00 | Reference | |
| Yes | 6 | 0.72 | (0.28–1.87) | 0.49 |
| **Azathioprine** | | | | |
| No | 54 | 1.00 | Reference | |
| Yes | 2 | 0.33 | (0.08–1.48) | 0.10 |
| **IVIG** | | | | |
| No | 47 | 1.00 | Reference | |
| Yes | 9 | 1.11 | (0.44–2.80) | 0.83 |
| **Cyclophosphamide** | | | | |
| No | 50 | 1.00 | Reference | |
| Yes | 6 | 1.06 | (0.35–3.23) | 0.92 |
| **Rituximab** | | | | |
| No | 48 | 1.00 | Reference | |
| Yes | 2 | 0.09 | (0–∞) | 0.37 |
